# Vision screening and refraction of Greenlandic schoolchildren

**DOI:** 10.1111/aos.16740

**Published:** 2024-07-14

**Authors:** Nick Duelund, Ivan Nisted, Marit Eika Jørgensen, Steffen Heegaard, Hanne Jensen

**Affiliations:** ^1^ Queen Ingrids Healthcare Center Nuuk Greenland; ^2^ Greenland Center of Health Research, Institute of Health and Nature Ilisimatusarfik University of Greenland Nuuk Greenland; ^3^ Department of Ophthalmology Rigshospitalet Copenhagen Denmark; ^4^ University of Copenhagen Copenhagen Denmark; ^5^ Danish College of Optometry and Vision Science Dania Academy Randers Denmark; ^6^ Department of Clinical Medicine Aarhus University Aarhus Denmark; ^7^ Steno Diabetes Center Greenland Nuuk Greenland; ^8^ University of Southern Denmark Odense Denmark

**Keywords:** amblyopia, Arctic, child, refraction, screening, visual acuity

## Abstract

**Purpose:**

To estimate the prevalence of amblyopia and refractive errors among 6‐year‐old children in Greenland and to assess the impact of incorporating autorefraction, stereoacuity and near visual acuity testing into vision screening.

**Methods:**

In this cross‐sectional study, 517 children (238 girls and 279 boys) from 21 locations in Greenland were screened using HOTV charts for distance and near visual acuity (VA), stereoacuity test and non‐cycloplegic autorefraction. Referral criteria for further ophthalmological examination included a VA of ≥0.2 logMAR on the worse‐seeing eye or an interocular VA difference of ≥2 lines.

**Results:**

Initial screening identified amblyopia (defined as VA of ≥0.3 logMAR) in 7% (unilateral) and 3% (bilateral) of children. However, subsequent ophthalmological examinations confirmed amblyopia in under 40% of referrals. Significant interocular VA differences were found in 9%. The prevalence of refractive errors at the screening was 3% for myopia (≤−0.5 dioptres), 10% for hyperopia (>+2.0 dioptres) and 14% for astigmatism (≤−1.00 dioptres), while the corresponding prevalences at the ophthalmological examination were 4% for myopia, 8% for hyperopia and 6% for astigmatism. Combining screening measurements increased the positive predictive values, thereby enhancing screening accuracy. Specifically, the incorporation of autorefraction or stereoacuity with distance VA demonstrated to be the most effective combination. Six percent of the children were prescribed glasses after the screening procedure.

**Conclusion:**

This study provides the first visual profile of Greenlandic schoolchildren. Incorporating autorefraction, stereoacuity and near visual acuity in vision screenings enhanced the efficacy of detection of vision anomalies. Although this may lead to more false positives, accurate screening is crucial in regions with limited ophthalmological resources.

## 1 INTRODUCTION

The primary purpose of vision screening is the early detection of children with strabismus and refractive errors, which both increase the risk of amblyopia (WHO, 2019), with the latter being one of the most common causes of visual impairment globally (Flaxman et al., 2017).

In Greenland, vision screening for schoolchildren is recommended at the age of 6 years by a school nurse, following the Danish guidelines (Danish Health Authority, 2019; Government of Greenland, 2005). While high participation rates for vision screening have been reported in Scandinavia (Bro & Löfgren, 2023; Falkenberg et al., 2019; Høeg et al., 2015), the participation rate in Greenland remains unknown. Although the first vision screening for children in Greenland is recommended at the age of four, the participation rate at this initial stage remains unknown. However, the coverage rate of the measles, mumps and rubella vaccine, administered during the 4‐year health examination where vision screening is also performed, is as low as 40% in the districts (Albertsen et al., 2020). This suggests that the coverage for preschool vision screening is likely similarly low. The current referral criterion is distance visual acuity of ≥0.2 logMAR. Moreover, Greenland does not have a permanent ophthalmologist, relying on annual visits from Danish consultants to its 17 cities. Most ophthalmological examinations in Greenland are conducted via telemedicine, available in only nine cities across Greenland. Ophthalmological patients must be transported to the nearest city with equipment for the examination. Additionally, seven of 17 cities in Greenland lack school nurses, further limiting the availability of ophthalmological examinations.

Greenland's population consists of 56 000 inhabitants where >80% are Inuit (Statistics Greenland, 2024). The healthcare system is divided into five regions, each with a regional hospital; additionally, the Capital hosts the national hospital. All specialised care is available in the Capital. The regional hospitals operate primarily in a primary care setting.

The current vision screening method in Greenland is performed using the Østerberg picture chart (Osterberg, 1965), which has several limitations. These include a lack of equidistant size difference between lines with increasing number of figures and increasing distance between figures with increasing visual acuity demand. Hence, the crowding phenomena vary as the figures change size (Kaiser, 2009), and comparison with logMAR visual acuity charts indicates an underestimation of visual acuity, especially among patients with low vision (Yu et al., 2021), and inferior test–retest accuracy (Lovie‐Kitchin, 2015).

The introduction of preschool vision screening based on distance visual acuity is an important tool in reducing the prevalence of amblyopia (Høeg et al., 2015; Thorisdottir et al., 2019), but significant hyperopia, anisometropia and astigmatism may go undetected, suggesting that a more sensitive vision screening is required.

Visual impairment in children with amblyopia has multiple negative impacts on their daily activities. Amblyopic children exhibit a 25% slower reading speed compared to children without visual impairment, a deficit that persists even when the amblyopic eye is occluded (Kelly et al., 2023). Children successfully treated for strabismus, either with optical correction or surgery, no longer show reduced reading speed (Kelly et al., 2015). Additionally, amblyopic children demonstrate impaired motor function with reduced balance and manual dexterity (Birch et al., 2023; Brin et al., 2022). The quality of life is frequently reduced in children with residual amblyopia compared to non‐amblyopic children with glasses (Hatt et al., 2020), further stressing the need for early detection of visual impairment.

The prevalence of amblyopia and refractive errors among Inuit populations in Greenland and other circumpolar countries has not been studied in recent years. However, one recent study found that 5.9% of the children aged 0–9 years were at risk of developing amblyopia in Nunavik, Canada (Tousignant & Brûlé, 2022).

Hence, the aim of this study was to estimate the prevalence of amblyopia and refractive errors and to assess whether adding autorefraction and stereoacuity testing to the screening procedures will reduce the number of undetected vision anomalies among 6‐year‐old children in Greenland.

## 2 MATERIALS AND METHODS

### 2.1 Design

A population‐based cross‐sectional study covering 13 cities and 10 settlements in Greenland. The three northernmost cities and one on the east coast were not included due to the lack of school nurses. Three cities in South Greenland without school nurses were included, as the dispatch of a school nurse from the Capital made it possible to conduct the vision screenings. The study was conducted between September 2017 and October 2018. The study followed the Tenets of the Declaration of Helsinki and was approved by the Greenlandic Science Ethics Committee (ID‐number 2023‐15841) and the Greenlandic Health Service. In 2017, the standard vision screening protocol, which is part of the regular health examination for school children and previously consisted of visual acuity measurement for distance, was expanded to include three additional tests: autorefraction, near vision testing and stereoacuity testing.

### 2.2 Participants

All first‐grade primary schoolchildren in participating cities and settlements in Greenland, aged 6 years (range 5.0–7.4 years), were invited to participate in this study.

### 2.3 Screening procedures

Prior to screening, the school nurses attended a 2‐day training course with theoretical lectures and supervised clinical training by a paediatric ophthalmologist to ensure procedural uniformity. Subsequently, all necessary equipment was provided.

The screening procedure consisted of:

- Distance visual acuity (VA) was measured monocularly at 3‐m distance with uncrowded letter presentation of a HOTV test chart (Cat. No. 2014) with five optotypes (H, O, T or V) on each line. An eye patch covered the non‐examined eye, and the test was repeated under binocular viewing conditions.
- Near VA was measured at 40 cm binocularly with a crowded near HOTV test chart (Cat. No. 2017).
- Stereoacuity was measured at 40 cm with Lang II Test.
- Non‐cycloplegic autorefraction was measured using Plusoptix A12R (Plusoptix GmbH, Nuremberg, Germany).
- Ongoing outpatient contact with an ophthalmologist or optometrist was recorded, and optical correction prescribed and/or worn during screening.
- Additional observations about the vision or eye anomalies, including strabismus, were recorded.

During screening, the children wore their habitual glasses. All results were sent to a paediatric ophthalmologist. Referral to a local ophthalmologist for a full eye and vision examination was recommended if distance Presenting Visual Acuity (PVA) was equal to or higher than 0.2 logMAR (≤6/9.5 Snellen fraction) or a difference of monocular VA of ≥2 lines between the right and the left eye. The ophthalmological examinations potentially included distance Best Corrected Visual Acuity (BCVA), a slit lamp examination, Hirschberg test for strabismus, and a cover test for manifest and latent strabismus, as well as cycloplegic autorefraction and ophthalmoscopy. In Greenland, a cycloplegic refraction is performed using 1% Cyclopentolate, instilled at least twice at 10‐min intervals. Autorefraction is measured at least 30 min after the last drop. However, it is important to note that we had no influence on the ophthalmological examination conducted by the ophthalmologist as the child was referred to the standard healthcare system in Greenland. Glasses were prescribed based on an individual evaluation.

### 2.4 Definitions

Amblyopia was defined as visual acuity of 0.3 logMAR (6/12 Snellen fraction) or poorer in one or both eyes (Repka, 2020; Sandfeld et al., 2019). Significant refractive errors were defined as hyperopia exceeding +2.0 dioptres (D), at least −0.5 D of myopia, −1.0 D of astigmatism and 1.0 D of anisometropia.

Positive predictive value (PPV) was calculated by dividing the number of children with positive screening results (i.e. measures outside the above‐mentioned criteria) who received glasses (true positive) with the number of children with positive screening results (true and false positive).

### 2.5 Statistics

Children were divided into three groups for analysis: not referred (group 1), referred to an ophthalmologist but without having glasses prescribed (group 2) and referred and having glasses prescribed (group 3).

Normally distributed continuous data are presented as mean and standard deviation (SD), while non‐normally distributed data are presented as median and interquartile range (IQR).

Categorical data were tested using a non‐parametric Chi‐squared test or Fisher's exact test when required. Paired *t*‐test was used to compare non‐cycloplegic with cycloplegic refraction (non‐cycloplegic minus cycloplegic refraction).

The participation rate was determined using the number of 6‐year‐old children in 2017 and birthrates from 2011, as obtained from Statistics Greenland (Statistics Greenland, 2024).

A *p*‐value of <0.05 was considered statistically significant.

## 3 RESULTS

**FIGURE 1:**
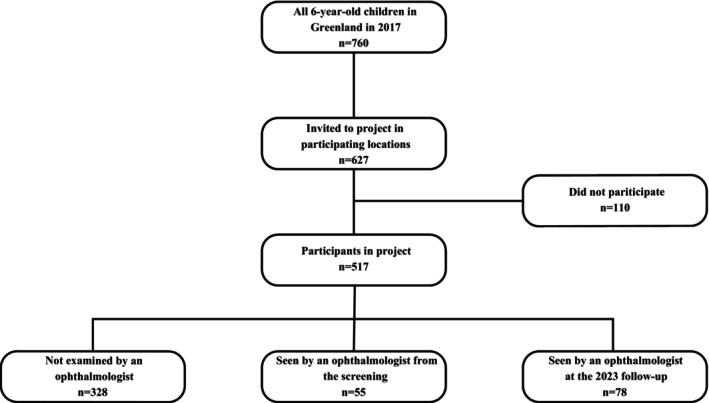
Flow chart of participants.

A total of 517 (238 girls and 279 boys) children participated in the standard health screening programme conducted by school nurses. Most children (93%) were from the cities, with the remaining from smaller settlements (Figure 1). The overall participation rate was 68% of the entire population of first‐grade children in Greenland, but as the three northernmost cities and one on the east coast were not included in the study, the participation rate among invited children was 82%.

**FIGURE 2:**
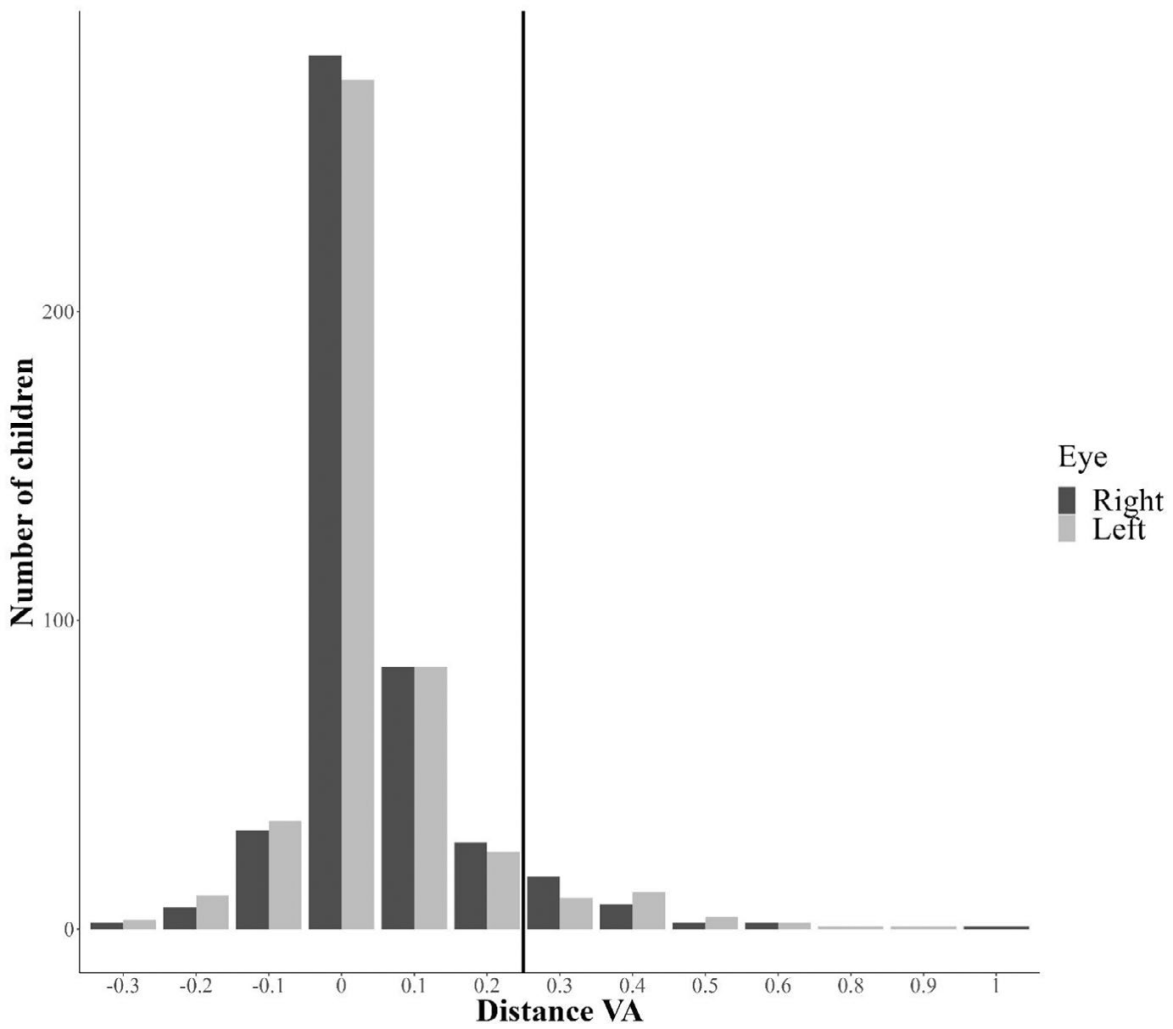
Distance visual acuity (VA) (logMAR) from screening of all Greenlandic children. The intercept line marks VA of 0.3 logMAR (6/12 Snellen fraction) or worse.

**FIGURE 3:**
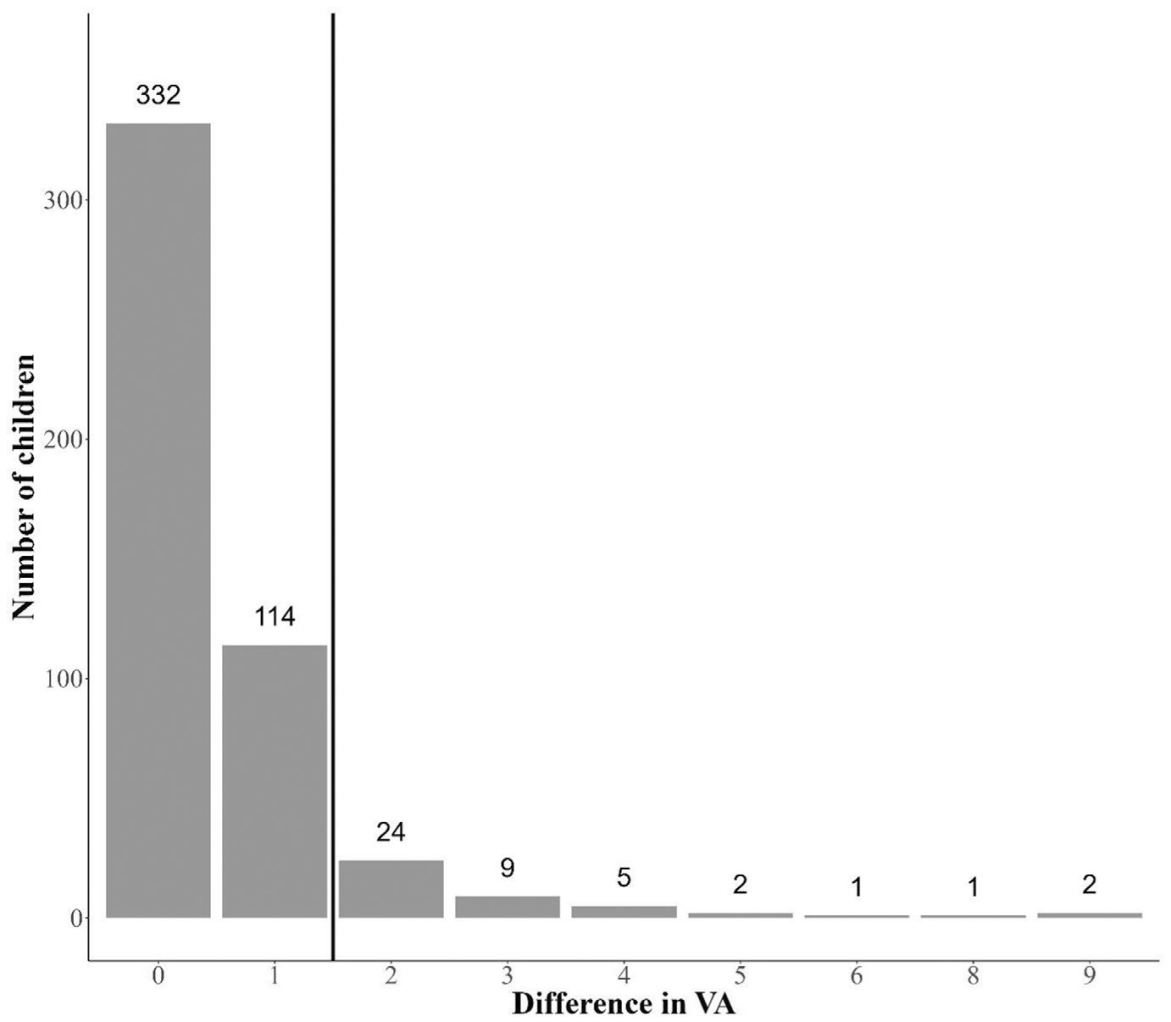
Number of Greenlandic children with interocular differences in visual acuity (VA) between the right and the left eye on the VA chart. The intercept line marks 2 or more lines in difference.

In total, 7% of the children (95% CI: 5%–9%) had amblyopia (VA equal to or higher than 0.3 logMAR, equivalent to 6/12 Snellen fraction) in either eye; and 3% (95% CI: 2%–5%) in both eyes (Figure 2) at the screening (PVA). After examination by a local ophthalmologist, the corresponding measurements with BCVA were 2% (95% CI 1%–3%) for either eye; and 1% (95% CI 0–3%) for both eyes. Forty‐four children (9% of all participating children) had an interocular VA difference (IOD) of two or more lines during the screening (Figure 3). Of these, 20 children had an IOD of three or more lines. Within the follow‐up period, five of these children continued to exhibit an IOD of three or more lines (BCVA). One had optic nerve anomaly, three had anisometropia of ≥3 D and one had astigmatism <−3.0 D. The remaining 15 children had the following outcomes: one child had a history of trauma to one eye; 11 children achieved normal BCVA in both eyes; one child had a BCVA of 0.2 in both eyes; one child was diagnosed with an anisometropia of 3 D and two lines of IOD; and one child's further information was unavailable.

At screening, 10% had hyperopia of >+2.0 D, 3% had myopia of ≤−0.5 D, 6% had anisometropi of ≥1.0 D, and 14% had astigmatism of ≤−1.00 D. The corresponding numbers at the ophthalmological examinations were 8% for hyperopia, 4% for myopia, 4% for anisometropia, and 6% for astigmatism. For the spherical equivalent refractions, the screening showed that 5% had hyperopia and 4% had myopia, while the ophthalmological examination revealed 6% had hyperopia and 4% had myopia.

Group 1 (462 children) were not referred to an ophthalmologist following the screening, group 2 (*n* = 24) were referred to an ophthalmologist, but were not prescribed glasses, and group 3 (*n* = 31) were referred to an ophthalmologist, and all were prescribed glasses.

Forty‐three children (8%) had ongoing outpatient contact with an ophthalmologist or optometrist at the time of screening, of which 18 already had glasses, while two did not wear their glasses during the screening.

Overall, the children exhibited a binocular VA of 0.01 logMAR, with the worse‐seeing eye at 0.07 and the better‐seeing eye at 0.02. The median stereoacuity was 200 seconds of arc. Additionally, the children generally had low hyperopia and low astigmatism, as detailed in Table 1.

**TABLE 1:** Data of all Greenlandic children's vision and refraction from screening and examination.

Measurements	All children *n* = 517
Distance VA screening (logMAR)	
Binocular	0.01 (0.10)^a^
Worse eye	0.07 (0.15)^a^
Better eye	0.02 (0.10)^a^
Interocular difference in VA^b^	0.0 [0.0, 1.0]^c^
Near binocular VA screening (logMAR)	0.01 (0.10)^a^
Lang II Test screening (seconds of arc)	200 arcsec [200, 200]^c^
Plusoptix refraction (D)	
Most hyperopic sph. eye	+1.17 (0.91)^a^
Least hyperopic sph. eye	+0.85 (0.82)^a^
Most astigmatic eye	−0.50 [−0.75, −0.25]^c^
Most hyperopic sph.eq. eye	+0.90 (0.81)^a^
Least hyperopic sph.eq. eye	+0.61 (0.75)^a^
Spherical equivalent right eye	+0.72 (0.79)^a^
Spherical equivalent left eye	+0.79 (0.78)^a^
Sph.eq. diff.	−0.07 (0.42)^a^
Cycloplegic refraction (D)	
Most hyperopic sph. eye	+1.97 (2.53)^a^
Least hyperopic sph. eye	+1.25 (2.47)^a^
Most astigmatic eye	−0.75 [−1.50, −0.25]^c^
Most hyperopic sph.eq. eye	+1.72 (2.53)^a^
Least hyperopic sph.eq. eye	+1.19 (2.68)^a^
Spherical equivalent right eye	+1.17 (2.74)^a^
Spherical equivalent left eye	+1.65 (±2.50)^a^
Sph.eq. diff.	−0.41 (1.44)^a^

Abbreviations: D, dioptres; sph., Spherical; sph.eq., Spherical equivalent; Sph.eq. diff., Spherical equivalent difference between the eyes; VA, visual acuity.

^a^ Mean (Standard Deviation).

^b^ Interocular difference in VA in number of lines.

^c^ Median [25% and 75% Interquartile].

There was a significantly higher proportion of children who were prescribed glasses (group 3) compared to children who were not prescribed glasses (groups 1 and 2), with a PVA for distance ≥0.3 logMAR (*p* = 0.03, χ^2^‐test) and an interocular difference in VA >2 lines (*p* = 0.003, Fisher's exact test). The two referred groups (groups 2 and 3) had a significantly higher proportion of abnormal Plusoptix measurements compared to the non‐referred group (group 1), whereas no difference between the two referred groups was found. Of the 462 children in group 1, 17 had a PVA for distance of ≥0.3 logMAR. Among these, six had ongoing contact with an ophthalmologist or an optometrist, and two had a new vision screening soon after, which was normal. Within the follow‐up period, seven children were seen by the local ophthalmologist, and glasses were prescribed to four of them. Two children who had not been examined by an ophthalmologist exhibited a visual acuity (VA) of 0.3 in the worse‐seeing eye (with the better‐seeing eye at 0.2 and 0.1, respectively). One child presented with mild hyperopia of +1.0, while the other had hyperopia of +2.5 and astigmatism of −1.5, as measured on the Plusoptix. Both children demonstrated normal near VA and stereoacuity (Table 2).

**TABLE 2:** Number and proportion of Greenlandic children with specific findings within the three groups.

	Group 1 *N* = 462 (%)	Group 2 *N* = 24 (%)	Group 3 *N* = 31 (%)	*p*‐value	*p*‐value group 2 versus group 3
VA					
Distance ≥0.3 worse eye	17 (4)	11 (46)	22 (71)	**<0.001** ^1^	**0.03** ^1^
Near ≥0.3 binocular	8 (2)	3 (13)	4 (13)	**<0.001** ^2^	1.0^2^
≥2‐line diff. in VA	19 (4)	3 (13)	15 (48)	**<0.001** ^2^	**0.003** ^2^
Plusoptix					
Abnormal^a^	91 (20)	13 (54)	15 (48)	**<0.001** ^1^	0.71^1^
No Plusoptix measurements	55 (12)	1 (4)	13 (42)		
Lang II test >400 arcsec	12 (3)	4 (17)	8 (26)	**<0.001** ^2^	0.35^2^

*Note*: Dioptres in spherical/cylindrical. The bolded values denotes statistical significance with *p* < 0.05.

Group 1: Not referred.

Group 2: Referred but not prescribed glasses.

Group 3: Referred and prescribed glasses.

Abbreviations: D, dioptres; diff., difference; VA, Visual Acuity (logMAR).

^a^ Hyperopia >+2.00 Dioptres (D), myopia ≤−0.50 D, astigmatism ≤−1.00 D or anisometropia ≥1.00 D.

^1^ χ^2^‐test.

^2^ Fisher's exact test.

Of the 55 (11%) referred children from the screening, 31 (6%) were prescribed glasses. Of the 24 children who were referred but not prescribed glasses, 22 had normal vision upon re‐examination, one was lost to follow‐up, and one child, for reasons unknown, was not prescribed glasses despite the presence of anisometric amblyopia.

By grouping all children seen by an ophthalmologist, no significant difference was found between the non‐cycloplegic and the cycloplegic refraction, except a larger astigmatism was found in cycloplegia (Table 3).

**TABLE 3:** Non‐cycloplegic (Plusoptix) refraction versus Cycloplegic refraction of Greenlandic children seen by an ophthalmologist (*n* = 133).

Measurements	Mean difference	95% CI	*p*‐value^a^
Spheric right eye (D)	+0.11	−0.25, 0.47	0.55
Spheric left eye (D)	+0.21	−0.16, 0.59	0.26
Cylinder right eye (D)	−0.07	−0.25, 0.11	0.45
Cylinder left eye (D)	−0.22	−0.38, −0.06	**0.008**

*Note*: Mean difference calculated as non‐cycloplegic minus cycloplegic refraction. The bolded values denotes statistical significance with *p* < 0.05.

Abbreviations: 95% CI = 95% Confidence Intervals; D, dioptres in spherical/cylindrical.

^a^ Paired *t*‐test.

The positive predictive value (PPV) increased when combining multiple measurements compared to a single measurement, whereas the absolute number of children receiving glasses decreased when combining more than two measurements (Table 4).

**TABLE 4:** Positive predictive value (PPV) of screening methods.

	PPV
One measurement	
Distance VA ≥0.2 worse eye (logMAR)	0.40 (33/83)
≥2‐line difference in VA	0.43 (16/37)
Distance VA ≥0.2 worse eye (logMAR) & ≥2‐line difference in VA	0.52 (15/29)
Near VA ≥0.2 binocular (logMAR)	0.23 (7/31)
Abnormal Plusoptix^a^	0.41 (28/68)
Lang II Test >400 arcsec	0.50 (12/24)
Combining two measurements	
Distance VA ≥0.2 & Near VA ≥0.2 binocular	0.37 (7/19)
Distance VA ≥0.2 & Lang II Test >400 arcsec	0.64 (9/14)
Distance VA ≥0.2 & Abnormal Plusoptix^a^	0.57 (21/37)
Near VA ≥0.2 binocular logMAR & Lang II Test >400 arcsec	0.50 (3/6)
Near VA ≥0.2 binocular logMAR & Abnormal Plusoptix^a^	0.55 (6/11)
Lang II Test >400 arcsec & Abnormal Plusoptix^a^	0.67 (4/6)
Combining three measurements	
Distance VA ≥0.2 & Near VA ≥0.2 binocular & Lang II Test >400 arcsec	0.60 (3/5)
Distance VA ≥0.2 & Near VA ≥0.2 binocular & Abnormal Plusoptix^a^	0.67 (6/9)
Distance VA ≥0.2 & Lang II Test >400 arcsec & Abnormal Plusoptix^a^	0.75 (3/4)
Near VA ≥0.2 binocular & Lang II Test >400 arcsec & Abnormal Plusoptix^a^	1.00 (2/2)
Combining all four measurements	
Distance VA ≥0.2 & Near VA ≥0.2 binocular & Lang II Test >400 arcsec & Abnormal Plusoptix	1.00 (2/2)

Abbreviation: VA, Visual Acuity (logMAR).

^a^ Hyperopia >+2.00 D, myopia ≤−0.5 D, astigmatism ≤−1.00 D or anisometropia ≥1.00 D.

## 4 DISCUSSION

The present study is the first to describe the distribution of visual acuity, refraction and stereoacuity, and to estimate the prevalence of visual impairment in Greenlandic schoolchildren.

Although the study did not include children from four cities and their settlements, it still covered most of the large and small cities and settlements, accounting for 68% of all first‐grade children. This extensive inclusion across various regions supports the representativeness of our findings, despite the exclusion of the northernmost and one eastern smaller city. The participation rate of 82% is similar to the participation rate of health examinations where vision screening is conducted among 5‐year‐old children in Denmark (Michelsen et al., 2007). This further supports that the estimated prevalence of visual impairment represents the first‐grade Greenlandic schoolchildren.

The prevalence of monocular and binocular amblyopia verified at the ophthalmological examination was 2% and 1%, respectively, which falls in between the findings of previous studies from Denmark and Canada (Ross et al., 1977; Sandfeld et al., 2019). However, a more recent Canadian study reported a higher prevalence of amblyopia, possibly due to a different amblyopia definition (Drover et al., 2008). The tendency for a lower prevalence of amblyopia in some studies may also be due to differences in methodology. By defining amblyopia as a VA of ≥0.3 logMAR and an interocular difference of ≥2 lines, the prevalence of monocular amblyopia in our study was 3% (15/517) at the screening and after the examination by the local ophthalmologist only 1% (5/517).

In the present study, the significantly higher estimated prevalence of amblyopia detected during screenings by school nurses, compared to examinations by ophthalmologists, may be attributed to the differences between PVA and BCVA or by learning effect, which reduces the false‐positive rate of amblyopia as found in previous studies (Sandfeld et al., 2018). Xiao et al. (2015) demonstrated how the prevalence of amblyopia varies with different definitions. The highest prevalence is observed when defining amblyopia as BCVA ≥0.3 logMAR (approximately 3%), while the lowest was seen using The Refractive Error Study in Children's suggested criteria (<1%) (Xiao et al., 2015). In the context of Greenland, determining the appropriate criteria for treating amblyopia is critical. If a child's BCVA is at 0.3 logMAR, the decision to forgo patch treatment should be carefully considered. Given the scarcity of school nurses and ophthalmologists in Greenland, establishing a cut‐off that identifies the majority of children who require ophthalmological evaluation and treatment is essential. Setting the amblyopia definition at ≥0.3 logMAR could effectively address this need.

Furthermore, significant differences were found between different ethnic groups using the same examination protocol, with lower prevalence of amblyopia for African, Nepali, Malay and Indian children, but not for Chinese and Hispanic children (Xiao et al., 2015), suggesting that both genetics and environmental factors impact the prevalence of visual impairment.

In this study, the prevalence of myopia (≤0.50 D) was 4% (95% CI 2%–6%), which is lower than in Canada (Yang et al., 2018), but higher than in Scandinavia (Bro & Löfgren, 2023; Sandfeld et al., 2018). This is similar to indigenous, but not non‐indigenous people from Australia (Hopkins et al., 2016). Such diverging results further support the impact of both genetics and environment on the development of ametropia, indicating that direct comparison of prevalence should be done with caution. This is further supported by the fact that compared to our study, hyperopia and anisometropia were less frequent in indigenous compared to non‐indigenous people, and the opposite was true for astigmatism (Hopkins et al., 2016). The prevalence of anisometropia and astigmatism found in our study corroborates with findings from Denmark, while hyperopia was lower (Sandfeld et al., 2018). No studies from other Indigenous people from the circumpolar regions have been performed.

Our results show that not all children in need of ophthalmological care or evaluation would be identified by standard distance visual acuity screening alone. This implies that adding other clinical assessments to the screening protocol would increase the efficacy. As Plusoptix autorefraction appears to be highly correlated with cycloplegic refraction, and by combining distance visual acuity screening with results from Plusoptix autorefractor, it is possible to identify children with hyperopia, myopia, astigmatism and anisometropia as shown in previous studies (Li et al., 2023; Silverstein & McElhinny, 2020). Adding stereoacuity and near visual acuity testing may further reduce the proportion of children with untreated vision anomalies, but the potential value of Plusoptix, stereoacuity and near visual acuity testing is most likely underestimated in the present study as only distance visual acuity was used as referral criterion. In our study, combining distance VA measurement with the Plusoptix autorefractor or stereoacuity appears to be the most effective combination. However, adding multiple tests to a vision screening will likely result in a higher number of false positive referrals for ophthalmological examination, thereby increasing costs and possibly delaying treatment of acute eye conditions. Retesting children who fail the initial screening before referring them to an ophthalmologist will most likely decrease false positive rates (Gyllencreutz et al., 2019).

An effective vision screening programme is crucial for identifying children with refractive errors and visual impairments, which can significantly impact their daily lives and overall quality of life. Previous studies have shown that appropriate corrective measures, such as glasses, can substantially improve academic performance and general well‐being (Kumaran et al., 2015; Pirindhavellie et al., 2023). However, wearing glasses may also have adverse effects, particularly in the context of physical activities and sports. Additionally, the social perceptions associated with wearing glasses in school settings may negatively affect a child's self‐esteem and mental well‐being (Kumaran et al., 2015). It is therefore essential to consider both the physical and psychosocial dimensions of prescribing glasses to children as part of a comprehensive vision care strategy.

Although this study suggests that the efficacy of vision screening can be enhanced, the nature of the study design only permits calculation of positive predictive values, and thereby insufficient on deciding to change the present vision screening programme. A study in Greenland, in which all children undergo the new screening method followed by a comprehensive ophthalmological examination, including cycloplegic refraction, could provide further insights into this issue.

Despite improvements, some children still receive false positive test results. However, as Greenland has no permanent ophthalmologist, an accurate first‐time vision screening is essential. Children might wait for more than a year to be examined by a consultant ophthalmologist, thereby delaying amblyopia treatment, why a high sensitivity is more important than a high positive predictive value. The fact that one child with anisometropic amblyopia was not prescribed glasses highlights the need for new guidelines to standardise glasses prescriptions for all children by consulting ophthalmologists in Greenland. However, the results indicate that trained school nurses can successfully perform screenings using the HOTV chart, which has replaced the less accurate Østerberg chart as the standard visual acuity chart for school children in Greenland.

In conclusion, enhancing the utilisation of preschool vision screening for Greenlandic children is essential to identify children with amblyopia and other vision impairments, given the limited availability of eye examinations in Greenland. By combining multiple vision tests, the accuracy will be increased. Our study showed that school nurses can conduct vision screening for schoolchildren in Greenland. (Xiao et al., 2015).

## AUTHOR CONTRIBUTIONS

Research Design: ND, IN, MEJ, SH, HJ. Data Acquisition/Research Execution: ND, HJ. Data Analysis/Interpretation: ND, IN, HJ, SH. Manuscript Preparation: Original Draft: ND, IN, HJ Writing, Review & Editing: ND, IN, MEJ, SH, HJ.

## FUNDING INFORMATION

This study was funded by Synoptik Fonden.

## CONFLICT OF INTEREST STATEMENT

No conflicts of interest.
